# Disproportionality and Case-Based Evidence for a Possible Association Between Fluoroquinolones and Kounis Syndrome

**DOI:** 10.3390/ph19050771

**Published:** 2026-05-14

**Authors:** Milena Cmiljanić, Miloš Milosavljević, Filip Jovčić, Mladen Pavlović, Srdjan Stefanović

**Affiliations:** 1 Medicines and Medical Devices Agency of Serbia, 11000 Belgrade, Serbia; milenacmiljanic@gmail.com; 2 Department of Pharmacology and Toxicology, Faculty of Medical Sciences, University of Kragujevac, 34000 Kragujevac, Serbia; 3 Department of Pharmacy, Faculty of Medical Sciences, University of Kragujevac, 34000 Kragujevac, Serbia; filipjovcic22@gmail.com (F.J.); sstefanovic@fmn.kg.ac.rs (S.S.); 4 Department of Surgery, Faculty of Medical Sciences, University of Kragujevac, 34000 Kragujevac, Serbia; drmpavlovic@gmx.com

**Keywords:** Kounis syndrome, fluoroquinolones, pharmacovigilance, VigiBase, adverse drug reactions, Bradford Hill criteria

## Abstract

**Background/Objectives**: Drug-induced hypersensitivity and cardiotoxicity are important yet often underrecognized clinical concerns, and fluoroquinolones are widely used antibiotics with well-documented safety issues. Given the limited systematic evidence and underreporting in pharmacovigilance databases, this study explored a potential association between fluoroquinolones and Kounis syndrome (KS) and the possibility of a class-related effect. **Methods**: This retrospective descriptive study analyzed individual case safety reports from VigiBase, complemented by published case reports to capture additional cases not recorded in the database. All fluoroquinolones reported as suspected drugs in KS cases were included, and a systematic search of major literature databases was undertaken to identify further case-level evidence. Quantitative data were explored using VigiLyze, while qualitative clinical data were extracted for case characterization. Literature cases underwent Naranjo assessment, and the overall body of evidence was evaluated using a qualitative Bradford Hill framework. **Results**: A descriptive disproportionality signal for fluoroquinolones was identified in VigiBase (IC_025_ = 1.3). Seventeen cases of fluoroquinolone-associated KS were identified across VigiBase and the published literature, all originating from unsolicited sources. Most cases involved ciprofloxacin and levofloxacin, whereas other fluoroquinolones were only rarely reported. Across cases, a consistent clinical pattern was observed, including a clear temporal relationship between drug exposure, allergic manifestations, and acute coronary events, compatible with hypersensitivity-mediated coronary involvement. **Conclusions**: KS cases associated with several fluoroquinolones were identified in pharmacovigilance data and the published literature, with the most consistent evidence observed for ciprofloxacin and levofloxacin. Although a class effect was not confirmed, a potential association cannot be excluded. These findings should be interpreted as hypothesis-generating, and further research is required to clarify underlying mechanisms, drug-specific risks, and clinical relevance.

## 1. Introduction

Kounis syndrome (KS), also referred to as allergic acute coronary syndrome, is a rare but clinically important condition characterized by simultaneous mast cell activation and coronary dysfunction following exposure to an allergen, most often a drug [1,2,3]. The underlying mechanism involves the release of mediators such as histamine, tryptase, leukotrienes, and various other inflammatory substances (e.g., prostaglandins, platelet-activating factor, cytokines, etc.), which may lead to coronary vasospasm, destabilization of atherosclerotic plaques, or stent thrombosis [4,5]. KS is currently classified into four types: type I (vasospasm without underlying atherosclerosis), type II (plaque rupture in pre-existing lesions), type III (stent thrombosis in the context of hypersensitivity), and type IV (hypersensitivity-induced events in patients with coronary artery bypass grafts) [6,7]. The clinical presentation of KS frequently mimics classic acute coronary syndrome (ACS), including chest pain, ST-segment elevation, and elevated troponins, but occurs in the setting of systemic hypersensitivity [5,7]. Due to its rarity and overlapping features with other cardiac conditions, KS remains underrecognized in clinical practice [2,8]. The most commonly reported triggers include medicines from groups such as nonsteroidal anti-inflammatory drugs, antibiotics, anticancer therapies, benzodiazepines, and serotonin receptor (5-HT_3_) antagonists, as well as insect bites [9,10,11].

Fluoroquinolones belong to the broader class of quinolone antibiotics, which were first introduced into clinical practice in 1961 [12]. Earlier, non-fluorinated quinolones such as nalidixic acid, pipemidic acid, and cinoxacin were among the first agents of this class but are no longer considered fluoroquinolones. In October 2018, the PRAC (Pharmacovigilance Risk Assessment Committee) of the European Medicines Agency (EMA) recommended restrictions on the use of fluoroquinolones following a review of serious, disabling, and potentially long-lasting adverse effects. These recommendations were endorsed by the CHMP, resulting in the suspension of certain quinolone antibiotics and a significant reduction in indications for the remaining fluoroquinolones in the EU [13].

A review of the available literature indicates that data on the association between fluoroquinolones and KS are limited to individual case reports, with no large epidemiological studies. In comparison with other drugs more frequently reported as triggers of this syndrome, fluoroquinolones are mentioned only in single case descriptions. The conclusion from the literature is that cases have been reported, but systematic data are lacking. The impetus for this study was the large number of reported cases of KS associated with different groups of antibiotics in the VigiBase database and in the published literature. Fluoroquinolones were selected for more detailed analysis because they are widely used in clinical practice to treat various bacterial infections, making them highly relevant from a public health perspective. Their safety profile has long been the subject of regulatory action, and in 2023, the EMA once again reminded healthcare professionals of the serious and potentially long-lasting adverse effects linked to this class of antibiotics [14]. Moreover, recent updates to product information for certain fluoroquinolones have included warnings about the possible occurrence of KS [15,16]. Taken together, these factors highlight the importance of examining fluoroquinolones in the context of a potential class effect related to KS. The aim of this study was therefore to explore the potential causal association between fluoroquinolones and KS, considering it as a possible class effect.

## 2. Results

### 2.1. Reports in the WHO Global Pharmacovigilance Database, VigiBase

A search of the VigiBase database identified 65 reported cases of KS associated with fluoroquinolones. After duplicates were removed using the VigiMatch algorithm, 56 unique cases were available for analysis. The period covered extended from 2010, when the first case was documented, through 1 February 2026, when the data collection concluded. These cases involved the following fluoroquinolones as suspect drugs: levofloxacin, ciprofloxacin, gemifloxacin, garenoxacin, and moxifloxacin. In addition, among the most frequently reported suspect or concomitant drugs were the following: hydroxyzine, clopidogrel, metronidazole, deflazacort, acetylsalicylic acid, alprazolam, piperacillin/tazobactam, as well as combinations of penicillins with beta-lactamase inhibitors. For the reported fluoroquinolones within the analyzed cases, marked differences were observed in reporting patterns. These differences related to the geographical origin of the reports, with the majority coming from Italy (20), Greece (8), Portugal (6), Spain (4), India (4), Turkey (4), and the United States (3), South Africa (3), Switzerland (1), Colombia (1) and Japan (1); the demographic characteristics of the patients, predominantly male (73.2%) and patients aged between 18 and 44 years; as well as the seriousness of the reported reactions, most of which were classified as serious (94.6%). The most frequently cited criterion for seriousness was caused/prolonged hospitalization (67.9%). Reports were most often submitted by healthcare professionals (69.6%). The highest number of reports was related to levofloxacin (30 cases), followed by ciprofloxacin (21 cases). Two cases were documented for gemifloxacin and moxifloxacin, while one case was reported for garenoxacin. Based on the disproportionality analysis presented in VigiBase, the following values were obtained for the fluoroquinolone group: IC_025_ = 1.3; PRR_025_ = 2.6; ROR_025_ = 2.6. For individual drugs, levofloxacin (IC_025_ = 1.4, PRR_025_ = 3.0, ROR_025_ = 3.0), ciprofloxacin (IC_025_ = 1.2, PRR_025_ = 2.6, ROR_025_ = 2.6), gemifloxacin (IC_025_ = −0.5, PRR_025_ = 5.7, ROR_025_ = 5.7), garenoxacin (IC_025_ = −2.3, PRR_025_ = 3.8, ROR_025_ = 3.8), and moxifloxacin (IC_025_ = −2.7, PRR_025_ = 0.2, ROR_025_ = 0.2). All 56 VigiBase cases were unsolicited reports, of which 54 originated from published literature, while two cases (one ciprofloxacin and one gemifloxacin) were reported through spontaneous reporting (Dataset A).

### 2.2. Adjudicated and De-Duplicated VigiBase Cases for Qualitative Synthesis

VigiBase reports were first reviewed, and an exclusion criterion based on a qualitative assessment was applied. Additional duplicates not previously recognized by the VigiMatch algorithm were removed, either because they had been reported multiple times from the same literature source or because they included more than one suspected drug.

A qualitative assessment of all 56 cases in VigiBase revealed that one ciprofloxacin case, initially reported as spontaneous, actually corresponded to a literature source [17]. Thus, the total number of literature-derived cases increased to 55; the only spontaneous case remaining was that of gemifloxacin. After duplicate removal, 4 cases of levofloxacin, 7 of ciprofloxacin, 1 of moxifloxacin, 1 of gemifloxacin, and 1 of garenoxacin remained, totaling 14 literature cases and 1 spontaneous case. Further analysis of all literature sources involved a detailed review to determine whether the fluoroquinolone was the sole suspect drug or whether another drug was also implicated. One ciprofloxacin case was excluded because it was reported together with gentamicin (both suspected) [18], leaving 6 ciprofloxacin cases. Four levofloxacin cases remained, while the single moxifloxacin case was excluded because it was reported in combination with deflazacort (both suspected) [19]. Gemifloxacin and garenoxacin each remained with one case. In the end, there were 6 ciprofloxacin, 4 levofloxacin, 1 gemifloxacin, and 1 garenoxacin cases from the literature, along with one spontaneous case of gemifloxacin (Dataset B).

### 2.3. Results of Literature-Derived Cases Not Reported in VigiBase

The literature was searched from database inception to 1 February 2026, leading to the identification of a total of four published cases of Kounis syndrome associated with fluoroquinolone antibiotics that were not reported in VigiBase. These comprised two cases involving ciprofloxacin [20,21] and two involving moxifloxacin [22,23] (Dataset C). No duplicate records were identified.

The systematic search of electronic databases identified four relevant case reports using broad search terms combined with Boolean operators. Two publications were retrieved via PubMed [21,23] and two via Google Scholar [20,22]. No eligible studies were excluded after full-text review due to duplication or insufficient clinical information.

### 2.4. Product Information Review

Beyond the literature search and case identification, regulatory and pharmacovigilance sources were also reviewed. The association of this syndrome was not documented in the online adverse effects database Martindale, but it was noted in the Summary of Product Characteristics (SPC) for levofloxacin approved by NAFDAC, the national regulatory authority of Nigeria [16]; moreover, since January 2026, the Coordination Group for Mutual Recognition and Decentralised Procedures—Human (CMDh) has recommended that information on KS be incorporated into the drug information for ciprofloxacin and moxifloxacin [15], while for gemifloxacin and garenoxacin such information was not found in SPCs approved by other regulatory authorities. These regulatory updates are reported to illustrate regulatory awareness and clinical relevance; however, such labeling decisions may be informed by the same pharmacovigilance and case-report evidence analyzed in this study and are therefore not considered independent evidence contributing to causal inference.

### 2.5. Bradford Hill—Final Combined Dataset

The final combined series prepared for the Bradford Hill assessment comprised a total of 17 cases, of which 16 originated from the literature and 1 from spontaneous reporting (gemifloxacin). Within this dataset, ciprofloxacin accounted for 8 cases, levofloxacin for 4 cases, gemifloxacin for 2 cases, moxifloxacin for 2 cases, and garenoxacin for 1 case. This consolidated dataset represents the basis for the Bradford Hill evaluation of fluoroquinolone-associated Kounis syndrome.

**Figure 1:**
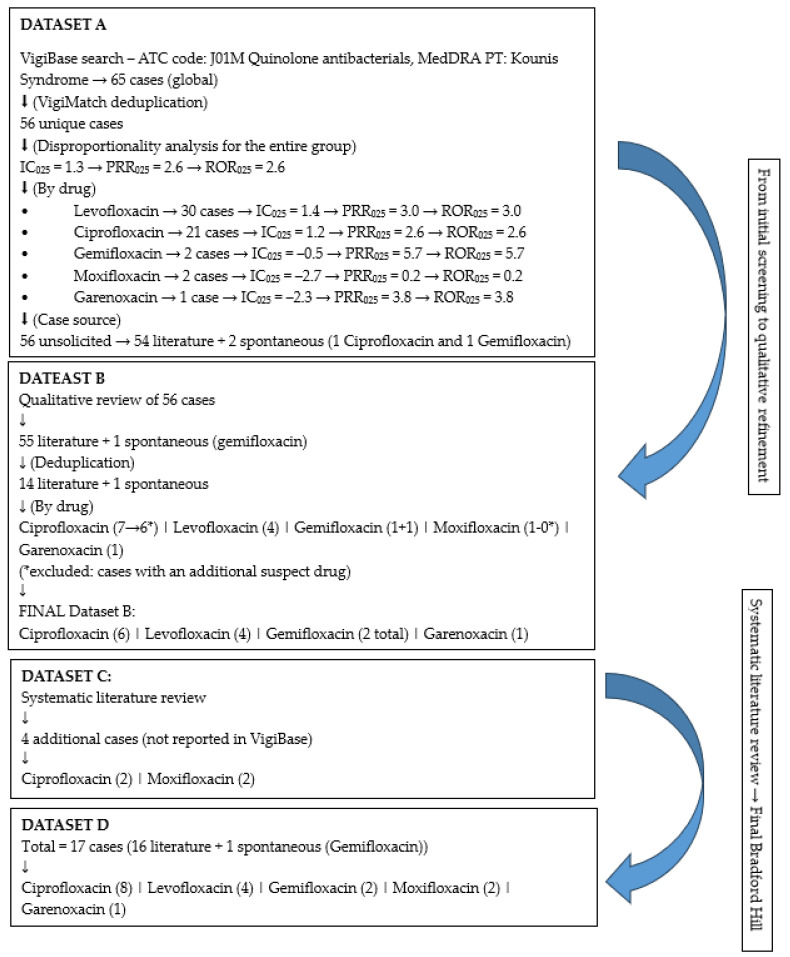
Flow diagram of dataset selection and integration for the Bradford Hill assessment of fluoroquinolone-associated Kounis syndrome.

The final combined series prepared for the Bradford Hill assessment comprised a total of 17 cases, of which 16 originated from the literature and 1 from spontaneous reporting (gemifloxacin). Within this dataset, ciprofloxacin accounted for 8 cases, levofloxacin for 4 cases, gemifloxacin for 2 cases, moxifloxacin for 2 cases, and garenoxacin for 1 case. This consolidated dataset represents the basis for the Bradford Hill evaluation of fluoroquinolone-associated Kounis syndrome (Dataset D). Datasets A, B, and C provided the foundation through initial VigiBase screening, qualitative assessment, and systematic literature review, and together they culminated in Dataset D, the final combined series used for the Bradford Hill assessment (Figure 1).

The clinical characteristics of the 17 identified cases are summarized below, organized by individual fluoroquinolone, beginning with ciprofloxacin.

In the ciprofloxacin-associated dataset, eight cases were described, involving patients aged between 30 and 85 years. Most patients had no prior history of coronary artery disease, except for one individual with a previous non-ST-elevation myocardial infarction and coronary stent implantation. Ciprofloxacin was administered orally or intravenously, most commonly for infectious conditions such as otitis media, urinary or urogenital tract infections, flu-like illness, gastrointestinal complaints, or perioperative prophylaxis. Allergic manifestations consistently preceded or accompanied the cardiac presentation, with cardiac symptoms occurring within minutes to hours depending on the route of administration and patient characteristics. Electrocardiographic findings consistently demonstrated ST-segment elevation, most frequently in the inferior leads. Coronary angiography revealed normal epicardial coronary arteries in seven cases, while one case was characterized by acute in-stent thrombosis. Kounis syndrome type I was identified in seven cases and type III in one case. Dechallenge was positive in all patients, no rechallenge was reported, and clinical outcomes were favorable.

In the levofloxacin-associated dataset, four cases were reported, involving patients aged between 28 and 68 years. Documented comorbidities included arterial hypertension, diabetes mellitus, hyperlipidemia, and nickel allergy, while two patients had no relevant medical history. Levofloxacin was administered orally, sometimes in combination with other antibiotics, for indications such as erysipelas, sinusitis, respiratory infection, or bronchopulmonary disease. Allergic manifestations preceded or accompanied the cardiac presentation, with time-to-onset ranging from 30 min to three days. Electrocardiographic findings were variable, and coronary angiography demonstrated significant lesions in some patients, whereas others had normal findings. Two cases were classified as Kounis syndrome type I, one as type II, and one patient experienced both type II and multiple recurrent type I episodes. Dechallenge was positive in all cases, with no rechallenge performed, except for one patient who subsequently tolerated ciprofloxacin following negative provocation testing. Management included corticosteroids, antihistamines, nitrates, antiplatelet agents, statins, ACE inhibitors, beta-blockers, oxygen therapy, and supportive measures, with intensive care admission and resuscitation required in severe cases. Clinical outcomes were favorable in all four patients.

In the moxifloxacin-associated dataset, two cases were identified. The first involved a 71-year-old woman who developed hypotension and chest pain approximately 30 min after intravenous administration and was diagnosed with type II Kounis syndrome presenting as non-ST-elevation myocardial infarction. The second case involved a 55-year-old male smoker with a history of aortic vasculitis who developed chest pain about 30 min after oral intake. Coronary angiography revealed severe left anterior descending artery stenosis, which was treated with stent implantation and balloon angioplasty. This presentation was classified as moxifloxacin-induced type II Kounis syndrome manifesting as an anaphylactic ST-elevation myocardial infarction. In both cases, dechallenge was positive, rechallenge was not performed, and clinical outcomes were favorable.

Cases of gemifloxacin-associated Kounis syndrome have been reported both in the literature and through spontaneous pharmacovigilance reporting in VigiBase. One published case involved a 46-year-old woman who developed chest pain, rash, palpitations, and dyspnea 15 min after the first oral dose of gemifloxacin and was diagnosed with type I Kounis syndrome, with complete clinical resolution. Additionally, a spontaneously reported case in VigiBase described a 33-year-old man treated for atypical pneumonia, in whom Kounis syndrome was reported as the sole adverse reaction and classified as serious. The reported time-to-onset was day 0, dechallenge was positive, rechallenge was not performed, and causality was assessed as probable/likely, with documented recovery.

Finally, one unsolicited garenoxacin-associated case retrieved from VigiBase was included and originated from a literature source. The case involved a 68-year-old woman and was classified as serious under the criterion of “other medically important condition.” Garenoxacin was administered orally at a dose of 200 mg once daily, with no indication or concomitant medications reported. Kounis syndrome was the only documented adverse event, with the outcome described as unknown. Neither dechallenge nor rechallenge was reported, the time-to-onset was unavailable, and no formal causality assessment was provided.

The characteristics of all 17 cases are summarized in Table 1.

**Table 2:** Bradford Hill criteria ratings for the association between fluoroquinolones and Kounis syndrome (drug level assessment).

Criteria	Ciprofloxacin	Levofloxacin	Moxifloxacin	Gemifloxacin	Garenoxacin
Strength of association	Signal consistent with an association	Signal consistent with an association	Hypothetical, based on isolated reports	Hypothetical, based on isolated reports	Hypothetical, based on isolated reports
Consistency	Signal consistent with an association	Signal consistent with an association	Hypothetical, based on isolated reports	Hypothetical, based on isolated reports	Not supported
Specificity	Uncertain/limited	Uncertain/limited	Hypothetical, based on isolated reports	Hypothetical, based on isolated reports	Not supported
Temporality	Signal consistent with an association	Signal consistent with an association	Hypothetical, based on isolated reports	Hypothetical, based on isolated reports	Not supported
Dose–response relationship	Not supported	Not supported	Not supported	Not supported	Not supported
Biological plausibility	Signal consistent with an association	Signal consistent with an association	Signal consistent with an association	Signal consistent with an association	Signal consistent with an association
Coherence	Signal consistent with an association	Signal consistent with an association	Signal consistent with an association	Signal consistent with an association	Not supported
Experimental evidence	Signal consistent with an association	Signal consistent with an association	Hypothetical, based on isolated reports	Hypothetical, based on isolated reports	Not supported
Analogy	Signal consistent with an association	Signal consistent with an association	Signal consistent with an association	Signal consistent with an association	Signal consistent with an association

## 3. Discussion

Applying the Bradford Hill framework across all fluoroquinolone antibiotics (ciprofloxacin, levofloxacin, moxifloxacin, gemifloxacin, garenoxacin), the following considerations emerged.

### 3.1. Strength of Association

The final combined series used for the Bradford Hill assessment comprised clinically well-documented cases, predominantly reported in the published literature, with ciprofloxacin and levofloxacin being the most frequently implicated drugs. This clinically consolidated case series constituted the sole basis for the Bradford Hill causality assessment of fluoroquinolone-associated Kounis syndrome.

Data from VigiBase, together with the qualitative assessment of reported cases and the systematic literature review, were used to support the overall evaluation; however, only the clinically well-documented cases were included in the formal Bradford Hill assessment (Table 1).

Evidence relevant to the Bradford Hill criterion of strength was explored by contextualizing the findings with disproportionality measures derived from VigiBase. A positive reporting signal was observed for fluoroquinolones at the class level (IC_025_ = 1.3), with levofloxacin and ciprofloxacin showing positive IC_025_ values accompanied by elevated PRR_025_ and ROR_025_ estimates (Dateast B). According to UMC methodology, IC_025_ > 0 indicates a statistical signal; however, as these measures were based on 56 VigiBase reports that included duplicate entries, they should be interpreted as reporting disproportionality rather than a quantitative measure of risk [35,36,37].

Importantly, a disproportionality signal was identified in VigiBase..

Disproportionality analyses were used solely as hypothesis-generating tools and do not establish definitive causality or provide estimates of incidence.

The base was used solely to indicate signal detection at the pharmacovigilance level and did not form the basis for defining the Bradford Hill criterion of strength. The assessment of strength was derived from the number and quality of clinically well-documented cases, while VigiBase findings served a supportive and contextual role.

### 3.2. Consistency

Across reported cases, fluoroquinolone-associated Kounis syndrome shows recurring descriptive clinical features, including rapid onset following drug exposure, a predominance of type I Kounis syndrome, and generally favorable outcomes. Taken together, these shared characteristics support descriptive consistency among reported cases rather than definitive statistical consistency.

Published literature indicates that Kounis syndrome has been reported across diverse age groups and geographic regions, with a higher frequency of reports from Southern Europe, particularly Turkey, Greece, Italy, and Spain, including regional clusters such as the Achaia district in Greece [38]. The distribution of Kounis syndrome subtypes described in these reports—type I in patients without underlying coronary artery disease, type II in those with pre-existing atherosclerotic disease, and type III in patients with coronary stents—closely parallels the subtype patterns observed among fluoroquinolone-associated cases included in this review [39,40].

#### 3.2.1. Ciprofloxacin

Across ciprofloxacin-associated cases, a recurrent descriptive clinical pattern was observed. Reactions occurred rapidly after exposure and most frequently manifested as type I Kounis syndrome, both in patients without documented comorbidities and in those with selected coexisting conditions. A single case of type III Kounis syndrome was reported in a patient with pre-existing coronary artery disease, which is consistent with the known pathophysiology of this subtype, while type II was not observed.

In the reported cases, allergic manifestations consistently preceded or accompanied the cardiac presentation. Coronary angiography demonstrated normal epicardial coronary arteries in all cases except the single type III presentation. Clinical outcomes were generally favorable, with recovery following drug discontinuation and appropriate management. Taken together, these observations support descriptive consistency with respect to timing, clinical presentation, Kounis syndrome subtype, and outcome across ciprofloxacin-associated cases.

#### 3.2.2. Levofloxacin

Despite variability in clinical severity across individual reports, all cases shared a consistent temporal relationship between levofloxacin exposure, the onset of allergic manifestations, and subsequent cardiac involvement. Clinical outcomes were favorable following drug discontinuation and appropriate management. Collectively, these observations support descriptive consistency across reported levofloxacin-associated cases, within the limitations inherent to a small number of observations.

#### 3.2.3. Moxifloxacin

The two available moxifloxacin-associated cases exhibited similar temporal characteristics, with symptom onset occurring approximately 30 min after drug exposure in both patients. In both instances, the clinical presentation was classified as type II Kounis syndrome, characterized by acute coronary syndrome in the presence of angiographically significant coronary artery disease.

Although the patients differed with respect to underlying conditions, both reports described a comparable sequence of drug exposure, allergic or inflammatory manifestations, and subsequent cardiac involvement. Clinical stabilization was achieved following drug discontinuation and appropriate management in both cases. Within the limits imposed by the small number of reported cases, these observations support descriptive consistency of clinical presentation and outcome in moxifloxacin-associated type II Kounis syndrome.

#### 3.2.4. Gemifloxacin and Garenoxacin

For gemifloxacin and garenoxacin, only isolated and sparsely documented cases have been reported, limiting any meaningful assessment of reproducible clinical or therapeutic patterns. One well-documented gemifloxacin case described type I Kounis syndrome in the absence of reported comorbidities, with rapid symptom onset and a favorable outcome following management of the allergic reaction. A second gemifloxacin report was identified through spontaneous reporting; however, it lacked detailed clinical characterization beyond the diagnosis of Kounis syndrome.

In contrast, the single available garenoxacin report did not specify the Kounis syndrome subtype and provided no information regarding clinical presentation, comorbidities, management, or outcome. Given the scarcity and limited quality of available data, consistency cannot be robustly assessed for either agent.

### 3.3. Specificity

Specificity considers whether a particular exposure is linked to a single, well-defined outcome. Hill acknowledged that many conditions have multiple causes and that specificity therefore represents a weaker causality criterion; however, when the same exposure is repeatedly associated with a similar clinical effect, the likelihood of an association is strengthened [41]. In the present analysis, specificity was considered intrinsically limited, as Kounis syndrome is a non-exclusive clinical entity that has been reported in association with a wide range of drugs and non-drug allergens [38,39].

For ciprofloxacin, most reports did not identify alternative competing triggers preceding symptom onset. In one case, cardiac manifestations followed treatment of an allergic reaction with adrenaline, which may have acted as a contributing factor; however, in the remaining reports ciprofloxacin was the only temporally associated exposure preceding both allergic and cardiac manifestations. This isolated instance of co-exposure modestly tempers exposure specificity.

For levofloxacin, published cases consistently attributed the reaction to the fluoroquinolone, with no clearly identified alternative triggers preceding symptom onset. Similarly, for moxifloxacin, both available cases were temporally associated with moxifloxacin exposure without documented competing triggers; nevertheless, the small number of reports and the presence of underlying coronary artery disease limit the assessment of exposure specificity.

For gemifloxacin, one well-documented case and one additional spontaneous report were identified; however, the limited number of observations and incomplete clinical characterization restrict reliable evaluation of specificity. For garenoxacin, the existence of a single poorly documented report—lacking information on timing, comorbidities, and clinical course—precludes meaningful assessment of specificity.

### 3.4. Temporality

Among the reported cases, fluoroquinolone exposure consistently preceded the onset of allergic manifestations and subsequent acute coronary events, fulfilling the temporal sequence required for considering an exposure–outcome relationship. In most reports, symptoms developed within a short time interval after drug administration, supporting a plausible temporal association.

For ciprofloxacin, symptom onset occurred within minutes to a few hours following administration, including immediate reactions after intravenous exposure and delayed presentations after oral intake. In all cases, allergic manifestations preceded or coincided with the development of Kounis syndrome. Even in the report where adrenaline was administered for anaphylaxis, ciprofloxacin exposure clearly preceded both the allergic reaction and the subsequent coronary event.

Levofloxacin-associated cases similarly demonstrated a clear temporal relationship, with allergic and cardiac symptoms developing within approximately 30–60 min after exposure in most instances, or recurring during continued therapy and resolving after drug discontinuation. For moxifloxacin, both reported cases showed acute coronary manifestations emerging approximately 30–60 min after drug administration, indicating a short and consistent latency period, albeit limited by the small number of observations.

In gemifloxacin-associated cases, the well-documented report described symptom onset within 15 min after the first dose, while the spontaneous report indicated same-day onset, supporting temporal precedence despite limited clinical detail. In contrast, for garenoxacin, the absence of reported time-to-onset data and incomplete clinical information preclude meaningful assessment of temporal relationship.

Across published reports, KS typically develops rapidly after exposure—most often within minutes to ≤1 h—although ranges from “immediately” up to several hours (and rarely longer) have been described across various triggers, including NSAIDs (e.g., diclofenac) [42], amoxicillin [43], insect bite anaphylaxis [44], and iodinated contrast media (~30 min) [45].

### 3.5. Dose–Response Relationship

For ciprofloxacin, levofloxacin, moxifloxacin, gemifloxacin, and garenoxacin, only minimal and fragmentary data regarding the dose and route of administration (oral or intravenous) were available across reported cases. These limited and inconsistently reported data do not allow assessment of a relationship between the administered dose and the severity or intensity of the reaction. Consequently, the dose–response criterion could not be reliably evaluated in the present analysis.

Importantly, Kounis syndrome represents an immunologically mediated hypersensitivity reaction that is not necessarily dose-dependent and may occur at any point during treatment, including after exposure to low drug doses. Such reactions are typically driven by IgE- or IgG-mediated immune mechanisms and alternative mast-cell activation pathways rather than cumulative or dose-related toxicity [46]. Accordingly, the absence of demonstrable dose–response relationships does not substantially weaken the overall causality assessment in this context.

### 3.6. Biological Plausibility

Hypersensitivity and anaphylaxis are mediated primarily through mast cell activation, most commonly via IgE-dependent mechanisms, leading to rapid degranulation and release of inflammatory mediators such as histamine, leukotrienes, prostaglandins, platelet-activating factor, and tryptase. Sensitization occurs when allergens are processed by antigen-presenting cells and induce Th2-driven immune responses, resulting in the production of allergen-specific IgE antibodies that bind to FcεRI receptors on mast cells and basophils. Upon re-exposure, IgE cross-linking triggers intracellular signaling cascades that promote mediator release and vascular effects, including increased permeability, edema, and vasospasm [47].

In addition to classical IgE-mediated pathways, hypersensitivity reactions may also arise through IgG-mediated immune mechanisms, complement activation, and IgE-independent mast cell stimulation. Notably, activation of the MRGPRX2 receptor on mast cells has been implicated in pseudo-allergic reactions to several pharmacological agents, including fluoroquinolones, leading to rapid mediator release in the absence of prior sensitization [48,49]. These pathways are particularly relevant in the cardiovascular system, where mast cell degranulation and downstream inflammatory signaling can precipitate coronary vasospasm, plaque destabilization, or thrombotic events.

Kounis syndrome represents an immunologically mediated intersection between hypersensitivity reactions and acute coronary events, driven by both IgE-dependent and non-IgE-mediated mast cell activation. In type I Kounis syndrome, allergic mediator release induces coronary vasospasm in angiographically normal arteries. In type II variants, inflammatory mediators and mast cell proteases such as tryptase and chymase contribute to destabilization and rupture of pre-existing atherosclerotic plaques, whereas type III Kounis syndrome involves hypersensitivity-associated thrombosis or restenosis of coronary stents through combined inflammatory and platelet-activating mechanisms [23,50,51].

Fluoroquinolones are well recognized triggers of immune-mediated adverse reactions, including immediate hypersensitivity and anaphylaxis. These reactions typically occur within hours of drug exposure and may be mediated either through classical IgE-dependent mechanisms or through direct, IgE-independent mast cell activation via MRGPRX2 [52,53,54,55,56]. Given the established ability of fluoroquinolones to induce mast cell degranulation and systemic allergic responses, these immunological pathways provide a biologically plausible mechanism by which fluoroquinolone exposure may precipitate Kounis syndrome.

Taken together, the known immunopathophysiology of hypersensitivity reactions, mast cell activation, and fluoroquinolone-induced allergic responses supports the biological plausibility of an association between fluoroquinolones and Kounis syndrome. However, this plausibility should be interpreted in the context of the predominantly case-based evidence and does not, by itself, establish definitive causality.

### 3.7. Coherence

In the presented cases, ciprofloxacin-, levofloxacin-, moxifloxacin-, and gemifloxacin-associated events were consistently characterized by allergic manifestations that preceded or accompanied acute coronary symptoms, in line with the established concept of immune-mediated coronary involvement in Kounis syndrome. Ciprofloxacin-associated cases encompassed all three variants of the syndrome, with type I occurring in patients with angiographically normal coronary arteries, type II in the presence of pre-existing atherosclerotic disease, and type III in association with coronary stent thrombosis. Levofloxacin-associated cases demonstrated a comparable pattern, with allergic prodromal symptoms followed by acute coronary events classified as type I or type II according to underlying coronary anatomy. Reported moxifloxacin-associated cases were limited to type II Kounis syndrome in patients with established coronary artery disease, whereas gemifloxacin-associated cases aligned with a type I phenotype characterized by allergic vasospasm and unobstructed coronary arteries. This distribution mirrors angiographic findings and the accepted clinical classification of Kounis syndrome, supporting descriptive coherence with established disease patterns [46].

Similarly, therapeutic approaches described in the literature—including H1/H2 antihistamines, corticosteroids, nitrates, antiplatelet and anticoagulant therapy, and supportive measures such as oxygen administration and coronary catheterization, with recognized caution regarding acetylsalicylic acid and beta-blockers—were consistent with management strategies applied across the reported cases [10,39,40]. Clinical responses observed following interventions targeting both allergic and coronary components further align with established treatment principles for allergic acute coronary syndromes.

Overall, published clinical observations of fluoroquinolone-associated Kounis syndrome are coherent with current clinical and regulatory knowledge, as reported patterns of presentation, management, and biological plausibility correspond to recognized allergic coronary mechanisms. However, this coherence should be interpreted descriptively and in the context of predominantly case-based evidence. Broader evidence suggests that some allergic acute coronary presentations may represent underrecognized forms of Kounis syndrome, warranting further investigation [57].

Notably, Kounis syndrome has been included in the Summary of Product Characteristics (SmPC) for ciprofloxacin [15], levofloxacin [16], and moxifloxacin [15], further supporting coherence with existing regulatory frameworks. Garenoxacin was not included in this coherence assessment because the available report lacked sufficient clinical, angiographic, and therapeutic detail to allow classification within established Kounis syndrome subtypes.

### 3.8. Experimental Evidence

For ciprofloxacin, eight cases were analyzed, the majority of which demonstrated a positive dechallenge, evidenced by clinical improvement following drug discontinuation; however, no intentional rechallenge was reported. For levofloxacin, four cases were available, all showing positive dechallenge without documentation of rechallenge. For moxifloxacin, two cases were reported with clinical stabilization after drug withdrawal, but without rechallenge data. For gemifloxacin, two cases were available, both with positive dechallenge and no information on rechallenge. For garenoxacin, a single case was reported, but without available data on dechallenge or rechallenge. Collectively, these observations indicate that Kounis syndrome symptoms generally resolve after discontinuation of the suspected fluoroquinolone, supporting a positive dechallenge pattern, while rechallenge data are largely unavailable across drugs. Positive dechallenge observed in spontaneous reports and case reports should be interpreted as supportive, but not definitive, evidence—particularly in the absence of systematic rechallenge data.

Experimental data show that fluoroquinolones have effects on the heart in different models. A study conducted in rats with induced acute myocardial injury, as well as in healthy animals, demonstrated that ciprofloxacin and levofloxacin cause dose-dependent changes in cardiac parameters, which are more pronounced in rats with acute myocardial injury than in healthy ones, accompanied by increases in serum cardiac enzymes and modulation in the expression of ion channels (Kv4.3, Kv1.2, Nav1.5) [58]. In an experimental study on anesthetized rabbits, it was shown that levofloxacin and the active metabolite of a new fluoroquinolone have lower potential compared to sparfloxacin and gemifloxacin, confirming that different fluoroquinolones have varying degrees of influence on cardiac electrophysiology. Furthermore, in a study on isolated Purkinje fibers from beagle dog hearts, fluoroquinolones were examined for their effects on action potential duration, and it was demonstrated that they alter the duration of the action potential and the QT interval, representing direct experimental evidence of their effects on the heart [59]. The Naranjo scale was applied descriptively; however, its applicability is limited for rare hypersensitivity-mediated cardiac syndromes and incompletely documented case reports. Naranjo scores were therefore interpreted cautiously as supportive, hypothesis-generating information rather than definitive causality evidence.

### 3.9. Analogy

From the perspective of analogy, it is noteworthy that other antibiotics—particularly beta-lactams [60,61,62] but also glycopeptides such as vancomycin [63]—have also been linked to cases of Kounis syndrome in clinical practice. These reports highlight that different antibiotic classes can trigger similar adverse reactions. In light of this, it becomes more plausible that fluoroquinolones, as another major group of antibiotics, may likewise contribute to allergic coronary events.

### 3.10. Limitations

Despite the strengths of integrating global pharmacovigilance data with a systematic literature review, several limitations must be considered when interpreting the present findings. Data from VigiBase are subject to well-recognized inherent limitations, including underreporting, selective reporting, duplicate entries, and incomplete clinical information. The quality of individual case safety reports (ICSRs) varies substantially, with many reports lacking key clinical details such as precise time-to-onset, comprehensive diagnostic evaluation, or confirmatory investigations (e.g., serum tryptase measurements).

A major limitation of this analysis is that the vast majority of identified cases were derived from the published literature. Following de-duplication and qualitative case-by-case verification, only a single unique spontaneous ICSR remained in VigiBase. Consequently, formal sensitivity analyses—such as exclusion of literature-derived cases to assess potential publication or notoriety bias, or restriction of analyses to reports submitted exclusively by healthcare professionals—were not feasible. For this reason, disproportionality estimates (IC_025_, PRR_025_, and ROR_025_), although generated within VigiLyze, could not be considered robust standalone quantitative signals and were interpreted descriptively at the level of signal detection rather than as measures of incidence or comparative risk.

The limited number of unique spontaneous cases also precluded meaningful stratified analyses by age, sex, geographic region, or reporter characteristics; therefore, demographic and clinical patterns are presented descriptively only.

In addition, disproportionate reporting for well-established fluoroquinolones such as ciprofloxacin may be influenced by notoriety bias, whereby increased clinical awareness and prior publications result in more frequent reporting compared with newer or less frequently prescribed agents. Confounding by indication or severity represents a further challenge, as fluoroquinolones are often prescribed for severe infections, sepsis, or systemic inflammatory states that may themselves precipitate cardiovascular stress or mimic acute coronary syndromes. Findings from a real-world comparative pharmacovigilance study using the FAERS database by Nakakura I. et al. [57] which identified quinolone use among other drugs, mortality, and polypharmacy as factors that increase the likelihood of reporting allergic coronary events not related to Kunis syndrome, support the possibility of the latter types of bias in the context of the problem of underdiagnosis of Kunis syndrome in daily clinical practice [57].

Kounis syndrome remains a diagnostic challenge in routine clinical practice and is likely underrecognized or misclassified, which may further affect the completeness and precision of pharmacovigilance data. As with all spontaneous reporting systems, the absence of reliable exposure denominators precludes estimation of true incidence or absolute risk.

Finally, additional literature-derived cases not captured in VigiBase further limited cross-source consistency. One reported case involved a preprint describing a possible moxifloxacin-associated type II Kounis syndrome that had not yet undergone peer review, while another published report could not be fully assessed due to lack of access to the complete article. Collectively, these limitations underscore that the present findings should be interpreted as hypothesis-generating and evaluated primarily in the context of qualitative case-level evidence rather than as definitive causal inference.

## 4. Materials and Methods

### 4.1. Study Design

This was a retrospective descriptive study based on individual case safety reports (ICSRs) retrieved from VigiBase. VigiBase, the World Health Organization’s global database of individual case safety reports (ICSRs), is maintained by the Uppsala Monitoring Centre [64]. The VigiBase data used for this study do not compromise the confidentiality or integrity of the reported adverse drug reaction cases. Approval for the analysis of data from this database was obtained from the Medicines and Medical Devices Agency of Serbia (ALIMS), and reference is made in this work to the “SaaS Agreement for UMC Products and Services offered to Members of the WHO Programme of International Drug Monitoring” from 2019. It serves as a key resource for identifying potential safety signals, including rare reactions such as KS. The analysis was further strengthened by incorporating relevant published literature. Initially, VigiBase was searched to identify fluoroquinolones that were reported as suspect drugs in cases of KS. All such fluoroquinolones were considered suspect, and for each of them, additional case reports were retrieved from the published literature to complement the database findings. By including both database-derived and literature-based cases, the study sought to comprehensively capture the spectrum of reported cases of KS (irrespective of type) associated with fluoroquinolone antibiotics, thereby enabling potential causality assessment based on the totality of available evidence. To systematically evaluate whether the observed associations indicate a possible causal relationship, the Bradford Hill criteria were applied.

The Bradford Hill criteria, introduced in the 1960s by epidemiologist Sir Austin Bradford Hill, provide a structured framework for moving from an observed association to a conclusion of causation. With his expertise in identifying links between drugs and health outcomes, Hill’s considerations have since been widely applied, often through manual review, to support causal inference in numerous epidemiological studies [65,66]. The main criteria for causality assessment include strength of the association, consistency, specificity, temporal sequence, biological gradient, biological rationale, coherence, experimental evidence, and analogy (strength refers to the magnitude of the association, often demonstrated through statistical tools such as disproportionality analysis; consistency is supported when similar findings are reported across different countries and populations; specificity highlights how closely the observed reaction can be linked to the suspected drug’s mechanism of action; a temporal sequence requires the adverse event to occur after the administration of the medicine within a plausible timeframe; biological gradient reflects whether the severity or frequency of the reaction increases with higher doses; biological rationale emphasizes the plausibility of the reaction based on pharmacological or biological mechanisms; coherence ensures that the observed data align with established medical knowledge; experimental evidence draws on results from clinical or preclinical studies that support or refute the association; and analogy offers other considerations [67,68].

The exposure of interest in this study was treatment with fluoroquinolone antibiotics, which were regarded as suspect drugs irrespective of the route of administration or other treatment characteristics. The primary outcome was the occurrence of KS following fluoroquinolone exposure, as identified in individual case safety reports from VigiBase and in published case reports.

### 4.2. Data Management and Analysis Approach

VigiBase represents the largest repository of individual case safety reports (ICSRs) worldwide, comprising more than 40 million reports contributed by over 180 countries and territories participating in the WHO Programme for International Drug Monitoring (status as of February 2025) [64]. Individual VigiBase ICSRs are subject to confidentiality and access restrictions and therefore cannot be publicly shared. To ensure transparency and reproducibility, aggregated disproportionality outputs and VigiLyze line-listing summaries generated for the present analysis are provided as Supplementary Materials.

All available global cases reported in VigiBase were included, with data extracted on 1 February 2026, which served as the data-lock date for this analysis. Data were analyzed using VigiLyze, the WHO signal management platform maintained by the Uppsala Monitoring Centre. A targeted search strategy was applied for antibiotics classified under ATC code J01M (fluoroquinolone antibacterials) and the adverse event Kounis syndrome, identified using the MedDRA Preferred Term “Kounis syndrome” (MedDRA version 28.1).

Exclusion criteria included duplicate reports and vaccine-related cases, implemented through application of the drugs-only filter. Only reports in which fluoroquinolone antibiotics were designated as suspect drugs were retained. Broader Standardised MedDRA Queries (SMQs) were deliberately not applied in order to maintain high specificity, given the distinct and well-characterized clinical presentation of Kounis syndrome. Following application of these criteria, both quantitative and qualitative datasets were extracted and exported from VigiLyze into structured Excel tables.

The quantitative dataset consisted of disproportionality metrics generated within VigiLyze using the standard VigiBase methodology, including the Information Component (IC), Proportional Reporting Ratio (PRR), and Reporting Odds Ratio (ROR), together with their corresponding lower confidence bounds (IC_025_, PRR_025_, and ROR_025_). In this framework, the reference set comprised all other reports in VigiBase excluding the specific drug–event combination of interest (“all other drugs” comparator). IC is a Bayesian disproportionality measure derived using the Bayesian Confidence Propagation Neural Network (BCPNN) and reflects the difference between observed and expected reporting frequencies. According to UMC methodology, a statistical signal is suggested when IC_025_ > 0 [69]. ROR and PRR were interpreted in conjunction with their lower confidence bounds (ROR_025_ ≥ 1 and PRR_025_ > 1, respectively) [70,71], with stability of estimates considered only when supported by a minimum number of reports—typically at least three, and in some analyses at least five [35].

The qualitative dataset included detailed case-level clinical information extracted from individual reports, encompassing patient demographics (age and sex), reporter type, geographic origin, suspect and concomitant medications, reported dose and route of administration, time-to-onset, dechallenge and rechallenge information, clinical outcome (e.g., recovered, fatal, unknown), seriousness, and available causality assessments. Case-level data mining and review were performed by M.C., who had authorized access to VigiLyze and prior experience with the platform.

### 4.3. Systematic Literature Search and Product Information Review

Following the identification of fluoroquinolones reported as suspected drugs in cases of Kounis syndrome (KS) within VigiBase, a systematic literature review was conducted to complement the database findings and identify published case-level evidence. Searches were performed in PubMed, Embase, Scopus, the Cochrane Library, and Google Scholar from database inception to 1 February 2026, using the keyword “Kounis syndrome” in combination with the international non-proprietary names (INN) of the fluoroquinolones of interest. To minimize the risk of missing relevant publications, broader terms such as “drug-induced Kounis syndrome,” “drug allergy,” “antibiotic allergy,” “drug anaphylaxis,” “antibiotic hypersensitivity,” and “acute coronary syndrome” were applied and combined using Boolean operators (AND, OR, NOT), without restrictions on language, publication type, or study design. Eligible studies included case reports or case series directly associating the fluoroquinolones of interest with Kounis syndrome and providing sufficient clinical detail to support case-level description. Exclusion criteria comprised review articles without original case-level data, reports with insufficient clinical information, and animal studies. Titles and abstracts were screened initially, followed by full-text assessment of potentially eligible publications. Conference abstracts were screened but were excluded when insufficient clinical detail was available. Preprints without peer review were considered on a case-by-case basis when detailed individual patient-level clinical data were provided. To identify additional relevant publications, reference lists of included studies and relevant reviews were manually screened.

From each eligible publication, data were extracted on patient demographics, comorbidities, time-to-onset (TTO), clinical presentation, diagnostic confirmation, management, and outcome. Study selection and data extraction were performed independently by two reviewers (MC and SS), with discrepancies resolved by discussion and consensus. To verify whether these associations were already recognized, the Summary of product characteristics and Product information of the fluoroquinolones identified in VigiBase and approved by major regulatory authorities (HPRA, MHRA, EMA, FDA, TGA, BfArM, etc.) were reviewed, together with reference sources such as Martindale: The Complete Drug Reference, in order to determine whether KS was documented in official product information. The review of product information was performed to assess regulatory recognition and clinical relevance and was not intended to provide independent confirmatory evidence of causality.

### 4.4. Case Assessment

For cases retrieved from VigiBase, the causality assessment provided within the database was reported. For cases identified through the literature review, causality was independently evaluated using the Naranjo adverse drug reaction probability scale [36]. Based on the Naranjo score, causality was categorized as definite (≥9), probable (5–8), possible (1–4), or doubtful (≤0) [37]. Finally, after integrating all available evidence from both sources, an aggregate-level assessment of the causal association was performed using the Austin Bradford Hill framework.

## 5. Conclusions

This study explored the potential association between fluoroquinolone use and Kounis syndrome by integrating global pharmacovigilance data with a systematic review of published case reports. Reports identified in VigiBase, together with qualitatively well documented cases from the literature, suggest a recurring clinical pattern compatible with hypersensitivity-mediated acute coronary events, particularly for ciprofloxacin and levofloxacin. However, a class-level pharmacovigilance signal does not imply risk equivalence across individual fluoroquinolones, and the absence of reported cases for specific agents should not be interpreted as evidence of absence of risk. The overall evidence base remains limited by the rarity of the event, the predominance of literature-derived cases, duplicate reporting, and the very small number of unique spontaneous reports available in VigiBase. Consequently, findings from disproportionality analyses should be interpreted at the level of signal detection and hypothesis generation rather than as evidence of definitive causality or quantification of risk. Taken together, the available data indicate that Kounis syndrome may represent a rare but clinically relevant hypersensitivity-associated complication of fluoroquinolone therapy. Clinicians should therefore remain vigilant for signs of allergic reactions accompanied by acute coronary symptoms when prescribing these agents. Further prospective studies, systematic registries, and well-characterized pharmacovigilance reports are needed to better define the incidence, risk factors, and underlying mechanisms of fluoroquinolone-associated Kounis syndrome.

## Figures and Tables

**Table 1:** Characteristics of included cases.

Case ID and Reference	Patient (Age/Sex)	Co-Morbidities	Indication	Dose/Route	Concomitant Medicines	KS Type	Time to Onset	Action Taken	Dechallenge Rechallenge	Outcome	Naranjo Causality
Cipro01 [20]	35 female	NR	Otitis media	NR	None	I	2 h 40 min	adrenaline, antihistamines, steroids, antiplatelets, statin, diltiazem	+/NR	Recovered	Possible
Cipro02 [24]	85 male	HTN, CKD4, Bladder cancer, follow-up, ex smoker, qs allergy	PAP	NR	Hydroxyzine Alprazolam	I	during infusion	antiplatelet, analgesia, UCA, long-acting nitrate	+/NR	Recovered	Probable
Cipro03 [25]	35 female	NR	uUTI	500 mg oral	NR	I	15 min	steroids, epinephrine, antihistamine, oxygen, aspirin, clopidogrel, heparin, UCA	+/NR	Recovered	Probable
Cipro04 [17]	71 male	HTN, previous NSTEMI	UTI	NR	ASA Clopidogrel Atorvastatin Carvedilol Omeprazole Valsartan	III	1–3 h	Adrenaline, noradrenaline, hydroxyzine, hydrocortisone, antithrombotics, PCI + stent	+/NR	Recovering	Possible
Cipro05 [21]	30 male	NR	Flu-like symptoms	oral	NR	I	Soon after administration	nitroglycerin, methylprednisolone, epinephrine, UCA, serum tryptase test	+/NR	Recovered	Probable
Cipro06 [26]	45 male	NR	Gastrointestinal infection	oral	NR	I	Soon after administration	i.v. diuretics, i.v. vasodilators, ECG, chest X-ray, laboratory tests, echocardiography, CA	+/NR	Recovered	Probable
Cipro07 [27]	39 male	Childhood exercise-induced asthma Allergic reaction to cefixim	Dysuria	oral	NR	NR	4 h	ECG, tenecteplase, ASA clopidogrel atenolol, rescue PCI, CA, prednisone, diphenhydramine, ranitidine, amlodipine, laboratory troponin tryptase IgE.	+/NR	Recovered	Probable
Cipro08 [28]	80 female	Hypothyroidism	Antibiotic prophylaxis	500 mg IV, single dose	Thyroxine	I	5 min	morphine, hydrocortisone, diphenhydramine, ranitidine IV, ECG, CA, Lab tests (troponin, cardiac enzymes, eosinophils, IgE)	+/NR	Recovered	Probable
Levo01 [29]	68 female	HTN, DM, HLD	Erysipelas	NR	NR	II	1 h	oxygen, methylprednisolone, albuterol, nitroglycerin, aspirin, clopidogrel, beta-blocker, statin, ACE inhibitor, CA, FFR testing, PCI, EC	+/NR	Recovered	Probable
Levo02 [30]	35 male	NR	Sinusitis	NR	NR	I	30 min	ECG angiography catheterization, stress test), skin tests, BAT, IgE, DPT	+/NR	Recovered	Probable
Levo03 [31]	58 female	Cutaneous nickel allergy, HTN	Respiratory infection	NR	Corticosteroids	I and II	soon after administration	CPR, CA, Intracoronary nitroglycerin, High-dose corticosteroids, antihistamines	+/NR	Recovered	Probable
Levo04 [32]	28 male	NR	Left bronchopulmonary focus (infection)	Oral levofloxacin 500 mg	piperacillin/tazobactam	I	30 min	ICU, ECG, supportive therapy	+/NR	Recovered	Probable
Moxi 01 [22]	71 female	HLD	CAP	0.4 g iv	Oseltamivir	II	30 min	UCA, IV dexamethasone, fluids, ASA, clopidogrel, atorvastatin, LMWH, cefuroxime, bronchodilators, antispasmodics	+/NR	Recovered	Probable
Moxi 02 [23]	55 male	Aortic vasculitis Smoker	Cough	NR	NR	II	30 min–60 min	ECG, UCA/PCI, ASA, clopidogrel, heparin, morphine noradrenaline, IV hydrocortisone, bronchodilators	+/NR	Recovered	Probable
Gem01 [33]	46 female	NR	URTII	320 mg oral	NR	I	15 min	ranitidine, diphenhydramine, methylprednisolone, saline, oxygen, salbutamol nebulizer, epinephrine, antihistamines, prednisolon, CA	+/NR	Recovered	Probable
Gem02	33 male		Atypical pneumonia	Oral, 1 tablet daily for 3 day	Tiotropium bromide, Formoterol fumarate		O days	UK	+/NR	Recovering	Probable
Gareno01 [34]	68 female	NR	NR	Oral, 200 mg once daily			NR	NR	NR/NR	NR	Doubtful

Abbreviations: KS—Kounis syndrome; HTN—high blood pressure (hypertension); Qs allergy—fluoroquinolone allergy; CKD4—stage 4 chronic kidney disease; UCA—urgent coronary angiography; UTI—urinary tract infection; uUTI—uncomplicated urinary tract infection; URTII—upper respiratory tract infection; CAP—community acquired pneumonia; DM—diabetes mellitus; PAP—perioperative antibiotic prophylaxis; NSTEMI—non-ST-elevation myocardial infarction; PCI—percutaneous coronary intervention; HLD—hyperlipidemia; ASA—acetylsalicylic acid; LMWH—low-molecular-weight heparin; BAT—basophil activation test; DPT—drug provocation test; CA—coronary angiography; FFR—fractional flow reserve; EC—echocardiography; ICU—intensive care unit; ECG—electrocardiogram; CPR—cardiopulmonary resuscitation; NR—not reported. Based on the integrated evidence, the aggregate-level causal association was further evaluated using the Austin Bradford Hill framework (Table 2).

## Data Availability

The original contributions presented in this study are included in the article/Supplementary Material. Further inquiries can be directed to the corresponding author.

## Supplementary Materials

The following supporting information can be downloaded at: https://www.mdpi.com/article/10.3390/ph19050771/s1, Table S1: Literature Search Strategy (Performed on 1 February 2026); Table S2: Eligibility criteria and screening process; Table S3: Manual review and handling of VigiBase cases after de duplication.
