# Supplementary material for: Disproportionality and Case-Based Evidence for a Possible Association Between Fluoroquinolones and Kounis Syndrome
Source: Pharmaceuticals (Basel). 2026 May 14;19(5):771. doi: 10.3390/ph19050771 (PMC13209738; doi:10.3390/ph19050771)
Supplement: Supplementary file 1 [file pharmaceuticals-19-00771-s001.zip › pharmaceuticals-4275923-supplementary.pdf]

Table S1. Literature Search Strategy (Performed on 1 February 2026)

Database	Search Query	Filters	Results
PubMed	(“Kounis syndrome”[MeSH Terms] OR “Kounis syndrome”[All Fields] OR “allergic myocardial infarction”[All Fields]) AND (“fluoroquinolones”[MeSH Terms] OR “ciprofloxacin”[All Fields] OR “levofloxacin”[All Fields] OR “moxifloxacin”[All Fields] OR “gemifloxacin”[All Fields] OR “garenoxacin”[All Fields])	None	(2)
Embase	('kounis syndrome'/exp OR 'kounis syndrome') AND ('fluoroquinolone derivative'/exp OR ciprofloxacin OR levofloxacin OR moxifloxacin OR gemifloxacin OR garenoxacin)	Emtree terms included	(0)
Cochrane Library	(“Kounis syndrome” AND (ciprofloxacin OR levofloxacin OR moxifloxacin OR gemifloxacin OR garenoxacin))	None	(0)
Google Scholar	“Kounis syndrome” AND (ciprofloxacin OR levofloxacin OR moxifloxacin OR gemifloxacin OR garenoxacin)	None	(2)
**Scopus**	TITLE-ABS-KEY ("Kounis syndrome" AND (fluoroquinolone OR ciprofloxacin OR levofloxacin OR moxifloxacin OR gemifloxacin OR garenoxacin))	None	(0)

Table S2. Eligibility criteria and screening process

Category	Description
Inclusion criteria	Case reports and case series reporting individual patient-level data with a direct association between fluoroquinolone exposure and Kounis syndrome.
Exclusion criteria	Review articles without original case-level data; animal studies; publications with insufficient clinical detail to support case description or assessment.
Publication types considered a priori	Full peer-reviewed articles; case reports and case series; correspondence or letters reporting original patient-level clinical cases.
Publication types excluded a priori	Conference abstracts without full clinical information.
Preprints	Preprints were assessed on a case-by-case basis and included only when detailed individual patient-level clinical data were available.
Screening procedure	Initial title and abstract screening followed by full-text assessment of potentially eligible publications; additional studies identified through manual screening of reference lists of included articles and relevant reviews.

Table S3. Manual review and handling of VigiBase cases after de-duplication

Step	Process description	Outcome / Rationale
Initial VigiBase retrieval	All Individual Case Safety Reports (ICSRs) reporting Kounis syndrome with suspected fluoroquinolones were retrieved from VigiBase.	Initial dataset consisted of 65 reports prior to formal deduplication.
Automated deduplication (VigiMatch)	VigiMatch was applied to remove duplicate reports based on probabilistic matching of key identifiers.	56 unique reports remained after automated deduplication.
Manual case-by-case review	All 56 de-duplicated VigiBase reports were manually reviewed case by case to verify report type, source, and underlying clinical information.	Manual review ensured accurate classification beyond automated processes.
Identification of spontaneous reports	During manual review, two reports were identified as unique spontaneous ICSRs: one involving gemifloxacin and one involving ciprofloxacin.	Confirmed existence of genuine spontaneous reporting independent of published case reports.
Identification of literature-derived VigiBase reports	Several VigiBase reports were found to correspond to published case reports, based on identical patient characteristics (age, sex), suspected and concomitant drugs, reactions, medical history, diagnostic tests, and procedures.	These reports were classified as literature-derived, not independent spontaneous cases.
Example of VigiBase–literature overlap	One VigiBase report (Safety Report ID: CO-INVIMA-300159757) initially classified as spontaneous was found to correspond exactly to a published case report.	Confirmed overlap with the publication by Navarro-Navajas et al., Medicina 2022;58:855, demonstrating duplication across sources.

Handling of overlapping VigiBase–literature cases	When a VigiBase report corresponded to a published case report, the case was counted only once and assigned to a single data source.	Prevented double counting and artificial inflation of case numbers.
Exclusion of multi-drug cases	VigiBase cases in which the suspected fluoroquinolone was reported together with another plausible suspected drug were excluded from qualitative synthesis.	Ensured clearer attribution for case-level qualitative assessment (Dataset B).
Definition of Dataset B	Dataset B comprised adjudicated, de-duplicated VigiBase cases retained after manual review and exclusion of overlapping and multi-drug reports.	Dataset B was used for qualitative case-level assessment.
Rationale for literature search strategy	Published cases already identified through VigiBase were not re-searched through bibliographic databases, as they were already captured and adjudicated within VigiBase.	Avoided redundant identification of the same cases through multiple sources.
Identification of additional literature-only cases	Bibliographic database searches (PubMed and Scopus) were used solely to identify additional published cases not present in VigiBase.	Resulted in four additional cases (two from PubMed and two from Scopus), which were included as literature-only cases.
Final case integration (Dataset D)	VigiBase-derived cases (after manual review) and literature-only cases were combined into a single final case series.	Dataset D represented the final combined dataset used for qualitative synthesis and Bradford Hill considerations, with each clinical case counted only once.
